# A last deglacial climate dataset comprising ice core data, marine data, and stalagmite data

**DOI:** 10.1016/j.dib.2018.11.008

**Published:** 2018-11-05

**Authors:** Zhi Liu, Shaopeng Huang, Zhangdong Jin

**Affiliations:** a School of Human Settlements and Civil Engineering, Xi’an Jiaotong University, Xi’an 710049, China; b Department of Earth and Environmental Sciences, University of Michigan, Ann Arbor, MI 48109-1063, USA; c State Key Laboratory of Loess and Quaternary Geology, Institute of Earth Environment, Chinese Academy of Sciences, Xi’an 710075, China

## Abstract

In this data article, a dataset of paleoclimatic records ranging from 22 to 9 thousand years before present is reported, which is related to the research article entitled “Breakpoint lead-lag analysis of the last deglacial climate change and atmospheric CO_2_ concentration on global and hemispheric scales” published in the journal of Quaternary International by Liu et al. (2018). In the dataset, 4 δ^18^O records derived from Greenlandic ice cores, 2 δD records and 7 δ^18^O records derived from Antarctic ice cores, 32 U^K’^_37_ records and 26 Mg/Ca records derived from marine deposits, and 17 δ^18^O records derived from cave stalagmites were collected and collated. General and statistical characteristics of these 88 proxy records are showed here. All of the data are stored in separate Microsoft Excel spreadsheets that are available for researchers.

**Specifications table**

**Table:** 

Subject area	Earth science
More specific subject area	Paleoclimatology
Type of data	Tables and Microsoft Excel
How data were acquired	Collected and collated from the website www.ncdc.noaa.gov/data-access/paleoclimatology-data/datasets and the paper of Shakun et al. [2]
Data format	Collated data
Experimental factors	None
Experimental features	None
Data source location	Globally distributed
Data accessibility	All of the data are with this article

**Value of the data**

• This is a dataset of 88 well-dated high-resolution proxy records compiled from 40 published papers.
• This dataset lays the foundation of the study of Liu et al. [1] on the lead-lag analysis of the last deglacial climate change and atmospheric CO_2_ concentration on global and hemispheric scales.
• This dataset can be used in further researches of data synthesis and regional comparison on various spatial and temporal scales over the last deglaciation.
• This dataset provides the potential to investigate the discrepancies of different paleoclimatic indicators, the interactions of Earth׳s different spheres, and the rules of the ice age termination from a global perspective.

## 1 Data

Tremendous efforts have been devoted to reconstruct the last deglacial climate history across the world; hence to integrate these records distributed in different geographical background is of necessary for the interpretation of the ice age termination from different spatial scales. In the original article [1], we published in the journal of Quaternary International, we collected and collated 88 well-dated high-resolution paleoclimatic records derived from ice cores, marine deposits, and stalagmites to composite global and hemispheric climate stacks. Here, this dataset is reported along with their statistical characteristics. Spatially, the sites of these records cover broadly the globe. Temporally, the average density over the period from 22 to 9 thousand years before present is 136 measurements per hundred years with a total of 17,699 data points. The general and statistical characteristics of these 88 records are showed in Table 1, Table 2, Table 3, Table 4, respectively.

**Table 1:** Statistical characteristics of the 13 ice core records in the dataset.

**#**	**Record**	**Proxy**	**Lat.**	**Long.**	**N**	**Min.**	**Max.**	**Mean**	**Chronology**	**Timescale ref.**	**Original ref.**
**1**	NGRIP	δ^18^O	75.1	−42.3	650	−44.97	−34.34	−40.09	GICC05	[3]	[3]
**2**	GISP2	δ^18^O	72.6	−38.5	816	−43.27	−34.12	−38.56	GICC05	[3]	[4]
**3**	GRIP	δ^18^O	72.5	−37.6	650	−42.72	−33.58	−38.76	GICC05	[3]	[5]
**4**	Renland	δ^18^O	71.3	−26.7	261	−31.89	−25.07	−28.36	GICC05	[3]	[6]
**5**	Law Dome	δ^18^O	−66.7	112.8	477	−28.99	−19.99	−24.44	GICC05	[3]	[7]
**6**	TALDICE	δ^18^O	−72.8	159.2	312	−41.95	−35.33	−38.29	AICC2012	[8]	[9]
**7**	EDML	δ^18^O	−75.0	0.1	864	−51.74	−41.68	−46.61	AICC2012	[8]	[10]
**8**	Dome Concordia	δD	−75.1	123.4	441	−448.00	−377.10	−412.97	AICC2012	[8]	[11]
**9**	Dome Fuji	δ^18^O	−77.3	38.7	52	−59.74	−53.18	−56.67	AICC2012	[8]	[12]
**10**	Taylor Dome	δ^18^O	−77.8	158.7	301	−44.76	−35.91	−38.91	Lemieux-Dudon	[13]	[14]
**11**	Vostok	δD	−78.5	106.8	199	−483.50	−425.30	−456.90	Lemieux-Dudon	[13]	[15]
**12**	Byrd	δ^18^O	−80.0	−119.5	307	−41.75	−32.36	−36.31	Lemieux-Dudon	[13]	[7]
**13**	Siple Dome	δ^18^O	−81.7	−148.8	387	−37.83	−26.16	−30.16	GICC05	[3]	[7]

Lat.=latitude; Long.= longitude; N represents the length of the record; Min. represents the minimum value in the record and Max. represents the maximum. All of these are the same as in Tables 2–4.

**Table 2:** Statistical characteristics of the 32 U^K′^_37_ records in the dataset.

**#**	**Record**	**Proxy**	**Lat.**	**Long.**	**N**	**Min.**	**Max.**	**Mean**	**Chronology**	**Timescale ref.**	**Original ref.**
**1**	W8709A-8	U^K′^_37_	42.5	−127.7	23	0.25	0.48	0.34	^14^C	[2]	[16]
**2**	PC-6	U^K′^_37_	40.4	143.5	57	0.36	0.58	0.46	^14^C	[2]	[17]
**3**	BS79-38	U^K′^_37_	38.4	13.6	38	0.40	0.67	0.50	^14^C	[2]	[18]
**4**	SU81-18	U^K′^_37_	37.8	−10.2	60	0.41	0.69	0.51	^14^C	[2]	[19]
**5**	MD95-2037	U^K′^_37_	37.1	−32.0	80	0.50	0.67	0.58	^14^C	[2]	[20]
**6**	M39-008	U^K′^_37_	36.4	−7.1	68	0.51	0.77	0.67	^14^C	[2]	[18]
**7**	MD95-2043	U^K′^_37_	36.1	−2.6	117	0.38	0.70	0.54	^14^C	[2]	[21]
**8**	MD01-2421	U^K′^_37_	36.0	141.8	62	0.48	0.72	0.59	^14^C	[2]	[22]
**9**	KT92-17 St. 14	U^K′^_37_	32.6	138.6	31	0.70	0.84	0.76	^14^C	[2]	[23]
**10**	MD98-2195	U^K′^_37_	31.6	129.0	83	0.68	0.91	0.75	^14^C	[2]	[24]
**11**	GeoB 5844-2	U^K′^_37_	27.7	34.7	41	0.60	0.92	0.83	^14^C	[2]	[25]
**12**	ODP 658C	U^K′^_37_	20.8	−18.6	93	0.58	0.73	0.68	^14^C	[2]	[26]
**13**	17940	U^K′^_37_	20.1	117.4	80	0.80	0.89	0.85	^14^C	[2]	[27]
**14**	74KL	U^K′^_37_	14.3	57.3	44	0.86	0.93	0.89	^14^C	[2]	[28]
**15**	M35003-4	U^K′^_37_	12.1	−61.3	36	0.85	0.94	0.90	^14^C	[2]	[29]
**16**	NIOP-905	U^K′^_37_	10.8	51.9	57	0.87	0.91	0.89	^14^C	[2]	[28]
**17**	MD02-2529	U^K′^_37_	8.2	−84.1	47	0.88	0.94	0.91	^14^C	[2]	[30]
**18**	MD01-2390	U^K′^_37_	6.6	113.4	54	0.90	0.97	0.93	^14^C	[2]	[31]
**19**	ME0005A-24JC	U^K′^_37_	0.0	−86.5	69	0.77	0.84	0.81	^14^C	[2]	[32]
**20**	V21-30	U^K′^_37_	−1.2	−89.7	36	0.83	0.88	0.85	^14^C	[2]	[33]
**21**	V19-28	U^K′^_37_	−2.4	−84.7	22	0.77	0.84	0.80	^14^C	[2]	[33]
**22**	GeoB 3910	U^K′^_37_	−4.2	−36.3	62	0.88	0.95	0.92	^14^C	[2]	[34]
**23**	GeoB 6518-1	U^K′^_37_	−5.6	11.2	52	0.77	0.87	0.82	^14^C	[2]	[35]
**24**	GeoB 1023-5	U^K′^_37_	−17.2	11.0	145	0.63	0.76	0.70	^14^C	[2]	[36]
**25**	MD79257	U^K′^_37_	−20.4	36.3	39	0.86	0.95	0.92	^14^C	[2]	[37]
**26**	GeoB 7139-2	U^K′^_37_	−30.2	−72.0	42	0.52	0.68	0.60	^14^C	[2]	[38]
**27**	MD03-2611	U^K′^_37_	−36.7	136.7	38	0.41	0.68	0.52	^14^C	[2]	[39]
**28**	MD97-2121	U^K′^_37_	−40.4	178.0	154	0.44	0.66	0.55	^14^C	[2]	[40]
**29**	ODP 1233	U^K′^_37_	−41.0	−74.5	138	0.32	0.58	0.47	^14^C	[2]	[41]
**30**	TN057-21-PC2	U^K′^_37_	−41.1	7.8	110	0.50	0.70	0.58	^14^C	[2]	[42]
**31**	SO136-GC11	U^K′^_37_	−43.5	167.9	73	0.36	0.62	0.48	^14^C	[2]	[43]
**32**	MD97-2120	U^K′^_37_	−45.5	174.9	109	0.26	0.53	0.37	^14^C	[2]	[40]

**Table 3:** Statistical characteristics of the 26 Mg/Ca records in the dataset.

**#**	**Record**	**Proxy**	**Lat.**	**Long.**	**N**	**Min.**	**Max.**	**Mean**	**Chronology**	**Timescale ref.**	**Original ref.**
**1**	MD01-2461	Mg/Ca	51.8	−12.9	131	1.19	3.25	1.97	^14^C	[2]	[44]
**2**	OCE326-GGC5	Mg/Ca	33.7	−57.6	35	0.61	1.58	0.91	^14^C	[2]	[45]
**3**	KNR140-51GGC	Mg/Ca	32.6	−76.3	36	3.34	4.91	4.03	^14^C	[2]	[45]
**4**	KY07-04-01	Mg/Ca	31.6	128.9	108	2.28	4.42	3.24	^14^C	[2]	[46]
**5**	MD02-2575	Mg/Ca	29.0	−87.1	45	2.50	4.33	3.37	^14^C	[2]	[47]
**6**	EN32-PC6	Mg/Ca	27.0	−91.3	81	2.94	5.06	3.96	^14^C	[2]	[48]
**7**	ODP 1144	Mg/Ca	20.1	117.6	43	2.75	3.92	3.16	^14^C	[2]	[49]
**8**	VM28-122	Mg/Ca	11.6	−78.4	41	3.15	3.99	3.62	^14^C	[2]	[50]
**9**	PL07-39PC	Mg/Ca	10.7	−65.0	132	2.87	4.89	3.68	^14^C	[2]	[51]
**10**	MD97-2141	Mg/Ca	8.8	121.3	178	3.24	4.87	4.08	^14^C	[2]	[52]
**11**	ME0005A-43JC	Mg/Ca	7.9	−83.6	59	3.07	4.60	3.69	^14^C	[2]	[53]
**12**	MD01-2390	Mg/Ca	6.6	113.4	56	3.47	4.97	4.09	^14^C	[2]	[31]
**13**	MD98-2181	Mg/Ca	6.3	125.8	230	3.55	6.12	4.53	^14^C	[2]	[54]
**14**	MD03-2707	Mg/Ca	2.5	9.4	121	2.83	4.55	3.56	^14^C	[2]	[55]
**15**	GeoB 4905	Mg/Ca	2.5	9.4	73	3.02	4.45	3.62	^14^C	[2]	[56]
**16**	TR163-22	Mg/Ca	0.5	−92.4	56	1.94	2.82	2.36	^14^C	[2]	[57]
**17**	V21-30	Mg/Ca	−1.2	−89.7	32	2.55	3.10	2.81	^14^C	[2]	[33]
**18**	GeoB 3129	Mg/Ca	−4.6	−36.6	121	3.23	5.05	4.22	^14^C	[2]	[58]
**19**	MD9821-62	Mg/Ca	−4.7	117.9	42	3.54	5.06	4.22	^14^C	[2]	[59]
**20**	MD98-2176	Mg/Ca	−5.0	133.4	92	3.73	5.66	4.56	^14^C	[2]	[54]
**21**	MD98-2165	Mg/Ca	−9.7	118.4	78	3.25	4.68	4.00	^14^C	[2]	[60]
**22**	MD98-2170	Mg/Ca	−10.6	125.4	35	3.82	5.74	4.59	^14^C	[2]	[54]
**23**	MD01-2378	Mg/Ca	−13.1	121.8	99	3.36	5.55	4.26	^14^C	[2]	[61]
**24**	ODP 1084B	Mg/Ca	−25.5	13.0	144	1.38	2.52	1.94	^14^C	[2]	[62]
**25**	KNR159-5-36GGC	Mg/Ca	−27.5	−46.5	32	2.92	3.91	3.41	^14^C	[2]	[45]
**26**	TN057-21	Mg/Ca	−41.1	7.8	92	1.13	2.45	1.56	^14^C	[2]	[63]

**Table 4:** Statistical characteristics of the 17 stalagmite δ^18^O records in the dataset.

**#**	**Record**	**Proxy**	**Lat.**	**Long.**	**N**	**Min.**	**Max.**	**Mean**	**Chronology**	**Timescale ref.**	**Original ref.**
**1**	Kesong Cave	δ^18^O	42.9	81.8	110	−12.03	−4.87	−10.40	^230^Th	[64]	[64]
**2**	Sofular Cave	δ^18^O	41.4	31.9	1091	−13.97	−8.97	−11.49	^230^Th	[65]	[65]
**3**	Fort Stanton	δ^18^O	33.3	−105.3	323	−10.53	−5.50	−7.47	^230^Th	[66]	[66]
**4**	Hulu Cave	δ^18^O	32.5	119.2	1382	−8.67	−4.03	−6.77	^230^Th	[67]	[67]
**5**	Jerusalem West Cave	δ^18^O	31.8	35.2	27	−5.84	−2.72	−4.06	^230^Th	[68]	[68]
**6**	Cave of the Bells	δ^18^O	31.8	−110.8	211	−11.24	−8.08	−9.68	^230^Th	[69]	[69]
**7**	Sanbao Cave	δ^18^O	31.7	110.4	580	−10.72	−6.20	−9.02	^230^Th	[70]	[70]
**8**	Soreq Cave	δ^18^O	31.5	35.0	109	−6.08	−2.73	−3.94	^230^Th	[71]	[71]
**9**	Yamen Cave	δ^18^O	25.5	107.9	1001	−9.98	−5.29	−8.15	^230^Th	[72]	[72]
**10**	Dongge Cave	δ^18^O	25.3	108.1	561	−9.34	−4.84	−7.52	^230^Th	[73]	[73]
**11**	Moomi Cave	δ^18^O	12.5	54.0	493	−3.66	0.37	−1.63	^230^Th	[74]	[74]
**12**	Northern Borneo	δ^18^O	4.0	114.8	695	−9.13	−6.08	−7.54	^230^Th	[75]	[75]
**13**	Liang Luar Cave	δ^18^O	−8.5	120.4	131	−5.68	−4.29	−4.94	^230^Th	[76]	[76]
**14**	Ball Gown Cave	δ^18^O	−17.0	125.0	129	−5.53	0.66	−2.91	^230^Th	[77]	[77]
**15**	Cold Air Cave	δ^18^O	−24.0	29.1	286	−4.41	−1.38	−2.96	^230^Th	[78]	[78]
**16**	Botuverá Cave	δ^18^O	−27.2	−49.2	76	−4.83	−1.52	−3.15	^230^Th	[79]	[79]
**17**	NW of the South Island	δ^18^O	−42.0	172.0	427	−3.71	−2.20	−2.92	^230^Th	[80]	[80]

## 2 Experimental design, materials and methods

The ice core data and stalagmite data included in this data article were collected from the website www.ncdc.noaa.gov/data-access/paleoclimatology-data/datasets, and the marine data of Alkenone ketone unsaturation index U^K′^_37_ and foraminifera Mg/Ca ratio were collected from existing research by Shakun et al. [2]. We extracted the data ranging from 22 to 9 kabp from each collected series and removed the vacant and duplicate values to constitute the dataset.
