# Supplementary material for: A last deglacial climate dataset comprising ice core data, marine data, and stalagmite data
Source: Data Brief. 2018 Nov 5;21:1764–70. doi: 10.1016/j.dib.2018.11.008 (PMC6249515; doi:10.1016/j.dib.2018.11.008)
Supplement: Supplementary file 1 — Supplementary material [file mmc1.docx]

**Conflict of Interest Form**

For data article “A last deglacial climate dataset comprising ice core data, marine data, and stalagmite data” by Zhi Liu, Shaopeng Huang, and Zhangdong Jin (DIB-D-18-02152).

We confirm that there are no conflicts of interest associated with this publication and there has been no significant financial support for this work that could have influenced its outcome.

We confirm that the manuscript has been read and approved by all named authors and there are no other persons who satisfied the criteria for authorship but are not listed. We further confirm that the order of authors listed in the manuscript has been approved by all of us.

We confirm that we have given due consideration to the protection of intellectual property associated with this work and that there are no impediments to publication, including the timing of publication, with respect to intellectual property. In so doing we confirm that we have followed the regulations of our institutions concerning intellectual property.

We understand that the Corresponding Author is the sole contact for the Editorial process. He is responsible for communicating with the other authors about progress, submissions of revisions and final approval of proofs.

Sincerely,

Shaopeng Huang

Zhangdong Jin

Zhi Liu
